# Association between lipid genetic and immunological status in chronically HIV-infected patients

**DOI:** 10.7448/IAS.17.4.19555

**Published:** 2014-11-02

**Authors:** Patricia Echeverría, Montse Guardiola, Marta González, Joan Carles Vallvé, Jordi Puig, Anna Bonjoch, Bonaventura Clotet, Josep Ribalta, Eugenia Negredo

**Affiliations:** 1 Fundació Lluita contra la Sida, Hospital Universitari Germans Trias i Pujol, Badalona, Universitat Autònoma de Barcelona, HIV, Barcelona, Spain; 2 Unitat de Recerca en Lípids i Arteriosclerosi, Institut d’ Investigació Sanitària Pere Virgili, Universitat Rovira i Virgili, Unitat de Recerca en Lípids i Arteriosclerosi., Barcelona, Spain

## Abstract

**Introduction:**

Polymorphisms in some host genes have a significant impact on susceptibility to HIV-1 infection and rate of disease progression [1, 2]. The purpose of the current sub-study was to find out the relationship between polymorphisms in genes involved in the lipid metabolism and the CD4/CD8 T-cell counts.

**Methods:**

Sub-study of a cross-sectional, observational study conducted in 468 patients with HIV infection attended at the outpatient clinic to investigate individual genetic predisposition to atherogenic dyslipidemia (AD). All patients were genetically characterized and all polymorphisms were in Hardy–Weinberg equilibrium. Thirteen polymorphisms were selected from nine genes: APOA5 (rs662799 and rs3135506); APOC3 (rs5128 and rs4520); LPL (rs328 and rs268); CETP (rs708272); HL (rs1800588); MTP (rs1800591); APOE (rs7412 and rs429358); LRP5 (rs7116604); and VLDLR (rs1454626). Lipid and lipoprotein parameters, CD4 and CD8 T-cell counts and plasma HIV-RNA were determinate. The statistical analysis was performed using SPSS statistical software version 19 (SPSS Inc., Chicago, IL, USA).

**Results:**

We studied 468 HIV-infected patients (men, 77%), with a mean (SD) age of 45.9 (19.7) years. The mean CD4 T-cell count and nadir CD4 was 547 (459) and 193 (159) cells/µL, respectively; 78.7% of participants were virologically suppressed. Patients carrying rs3135506 in the APOA5 gene presented a 9% increase in circulating TG levels (*p*=0.002) and 10% decrease in HDLc levels (*p*=0.005). Such association of APOA5 towards dyslipidemia was accompanied by a 21% decrease of the CD4 T-cell count (*p*=0.024) and a 19% increase in CD8 T-cell count (*p*=0.002) in carriers of the rare allele in the APOA5 rs662799 polymorphism adjusted by age and gender. Patients carrying the rare allele in rs5128 (APOC3) had a 16% decrease in circulating CD4 T cells (*p*=0.029); patients carrying rs1800591 (MTP) had a 29% decrease in CD4 T cells and 14% decrease in CD8 T cells (*p*=0.018 and *p*=0.008, respectively); patients carrying the rare allele rs1800588 in HL had a 11% increase in CD4 T cells (*p*=0.043); and carriers of the rs145626 in the VLDLR gene had 10% decrease in CD4 circulating T cells (*p*=0.013).

**Conclusion:**

Variants in genes involved in the development of AD may also influence the immunological host–virus equilibrium in chronically HIV-infected subjects [2, 3].
